# Citrus-Fruit-Based Hydroxyapatite Anodization Coatings on Titanium Implants

**DOI:** 10.3390/ma18051163

**Published:** 2025-03-05

**Authors:** Amisha Parekh, Alp Tahincioglu, Chance Walters, Charles Chisolm, Scott Williamson, Amol V. Janorkar, Michael D. Roach

**Affiliations:** Department of Biomedical Materials Science, University of Mississippi Medical Center, Jackson, MS 39216, USA; aparekh@umc.edu (A.P.); alptahincioglu@gmail.com (A.T.); chance23walters@gmail.com (C.W.); charles.chisolm22@gmail.com (C.C.); rwilliamson@umc.edu (S.W.);

**Keywords:** hydroxyapatite, carbonated apatite, tricalcium phosphate, Ca-release, Mg-release, titanium dioxide, anodization, osseointegration

## Abstract

The increasing demand for titanium implants necessitates improved longevity. Plasma-sprayed hydroxyapatite coatings enhance implant osseointegration but are susceptible to delamination. Alternatively, anodized hydroxyapatite coatings have shown greater adhesion strengths. The present study aimed to develop anodized hydroxyapatite coatings on titanium using commercial calcium-fortified fruit juice as a calcium source. Varying the electrolyte compositions enabled the formation of four oxide groups with different predominate calcium compounds. Each oxide’s morphology, crystallinity, chemistry, molecular structure, and adhesion quality were compared and contrasted. Nanoscale SEM images revealed a progression from porous surface oxide to white surface deposits to petal-like hydroxyapatite structures with the changing anodization electrolytes. Oxide thickness evaluations showed progression from a single-layered oxide with low Ca-, P-, and Mg-dopant incorporations to bi-layered oxide structures with increased Ca-, P-, and Mg-dopant incorporation with changing electrolytes. The bi-layered oxide structures exhibited a titanium-dioxide-rich inner layer and calcium-compound-rich outer layers. Furthermore, indentation analyses confirmed good adhesion quality for three oxides. For the predominate hydroxyapatite oxides, FTIR analyses showed carbonate substitutions indicating the presence of bone-like apatite formation, and ICP-OES analyses revealed prolonged Ca and Mg release over 30 days. These Mg-enhanced carbonated apatite coatings show much promise to improve osseointegration and future implant lifetimes.

## 1. Introduction

With humans living longer, the need to improve implants’ performance and effective lifetimes is constantly increasing. The global market for dental implants reached around $4.6 billion in 2019 [1], while the orthopedic implant market is expected to reach $9 billion by 2025 [2,3]. Osseointegration, defined as the structural and functional connection between the implant surface and the surrounding bone, serves as a physiological foundation for successful endosseous implantation [4]. However, inadequate bone–implant contact may result in fibrous scarring, leading to loosening at the interface between the bone and implant and subsequently causing implant failure [5]. Early dental implant failures are often associated with poor bone–implant contact [6]. Although dental implants have a high success rate (90–97%), the American Dental Association (ADA) reports that approximately 0.15–0.5 million patients in the United States still require revision surgeries yearly [7,8,9]. Moreover, aseptic loosening (impaired implant fixation) is the main reason for the failure of a prosthesis and is responsible for about 76% of prosthetic reoperations (50% of hip and 30% of knee prostheses) [10,11].

Titanium and its alloys are widely used as implant materials for making orthopedic and dental implants as a result of their high strength, strength-to-weight ratio, excellent corrosion resistance, and general biocompatibility. However, due to its bio-inertness, it quickly loses its bone–implant contact (BIC) [12,13,14]. Thus, titanium surfaces are often modified to improve their bioactivity. Sandblasting, acid etching, plasma spraying, and anodization are some of the commercially used titanium surface modification techniques and have achieved improved bone tissue reactions in vivo [15,16,17]. Anodization is an electrochemical process that can be used as a surface modification methodology to simultaneously modify the oxide coating surface roughness, porosity, crystallinity, and chemistry [18,19].

With the help of anodization, we can incorporate essential chemical elements into the oxide layer in one step. Calcium phosphates (CaP) are the main inorganic component in bone tissue and possess inherent biocompatibility. Thus, recent anodization studies have explored the addition of calcium (Ca) and phosphorus (P) into the titanium oxide layer [20,21,22,23,24,25,26,27]. Blood proteins adsorb onto the implant surface upon implant placement, and clot formation occurs. Ca ions help activate thrombin and fibrin formation and stabilize the clot, which facilitates the recruitment of bone-forming cells [28]. Ca ions have been known to play a critical role in the osseointegration of implants by regulating key proteins involved in osteoblast differentiation and bone regeneration, as evident from the results of a recent study showing that Ca-modified titanium surfaces showed early bone regenerative matrix formation, leading to faster osseointegration compared to unmodified surfaces [28,29,30]. Another study that created a Ca and hydroxyapatite-containing coating on titanium implants using an electrophoretic deposition process showed that the coated implants exhibited earlier bone development, mineralization, and maturation compared to uncoated implants in rabbit femurs [31].

Hydroxyapatite and Tricalcium phosphate are widely researched calcium phosphates and are routinely used in bone cements and bone substitutes [32]. Tricalcium phosphate has a well-known Ca/P ratio of 1.5 and has two phases, α-phase and β-phase [33,34]. Hydroxyapatite coatings have commonly been used on the surface of metallic implants to improve their bioactivity and to increase the BIC area [35]. Several methods, such as plasma spraying, sputtering, pulsed laser deposition, and sol-gel techniques, have been employed for the formation of hydroxyapatite coatings on titanium, and several reports have been published stating the improvement of osseointegration in this regard [35,36,37]. However, hydroxyapatite coating methods often suffer from the need for expensive specialized equipment, long preparation periods, and low adhesion strengths. One commonly used hydroxyapatite coating in clinical practice is formed by spraying hydroxyapatite particles onto the implant surface at high temperatures and then cooling rapidly [38]. These spray coatings bind easily to bone tissue but show a low bonding strength to the metal alloy substrate. Factors such as degradation of hydroxyapatite at high spraying temperatures and differences in the coefficient of thermal expansion (CTE) between hydroxyapatite and titanium substrate materials are the reasons for the low bonding strengths of the coatings [38,39]. Such low bonding strengths often lead to delamination at the coating-substrate interface, eventually leading to implant failure due to cracking and peeling of the coating [38,39].

Anodization as a surface modification technique is a simple processing method, is cost-effective, and improves coating adhesion and interfacial bonding [27]. A few research groups have recently explored anodization to form hydroxyapatite coatings on titanium implant surfaces [20,22,24,27]. Interestingly, hydroxyapatite-containing anodization coatings showed improved interfacial bonding and adhesion compared to plasma-sprayed hydroxyapatite coating counterparts [20,24]. Hydroxyapatite-containing anodized coatings have shown tensile bond strengths up to 44 MPa [26] and HV 0.3 microhardness values between 250 and 350 [24]. In our previous study, we produced magnesium-doped carbonated hydroxyapatite and tricalcium phosphate coatings on four titanium implant alloys in a single-step anodization process using an innovative citric-acid-based electrolyte [40]. Considering that citrus fruits and commercial fruit juices provide a natural source of calcium and phosphorus, in the present study, we explored citrus-based fruit juice as an alternative electrolyte calcium source component [41]. Thus, the present study aimed to develop calcium-releasing hydroxyapatite and tricalcium phosphate coatings using a novel citrus-fruit-based electrolyte composition implemented in a single-step anodization process. To the best of the knowledge of the authors, anodization in the citrus-fruit-based electrolytes to form hydroxyapatite coatings on implant materials has not been explored before.

## 2. Materials and Methods

### 2.1. Specimen Preparation

Commercially pure titanium grade 4 (CPTi4) discs were cut to 3 mm thickness from a 12.7 mm diameter bar stock. The CPTi4 discs were then briefly wet ground with 320 grit SiC paper to remove any rough edges generated during cutting, ultrasonically cleaned with laboratory detergent (Alconox^®^, White Plains, NY, USA), and rinsed with distilled water. The cleaned discs were then dipped in a nitric acid–hydrofluoric acid (10:1 ratio) solution (TURCO NITRADD, Henkel Corporation, Madison Heights, MI, USA) for 30 s to activate the surface for anodization.

### 2.2. Anodization

The citrus-fruit-based anodization electrolytes used are provided in Table 1. Each electrolyte consisted of a mixture of commercially available calcium-fortified orange juice (Calcium-Fortified Orange Juice, Kroger, Cincinnati, OH, USA), monobasic calcium phosphate (90%, Thermo Fisher Scientific, Waltham, MA, USA), and calcium acetate (99% Spectrum chemical, New Brunswick, NJ, USA). The calcium phosphate and calcium acetate powders were dissolved into the calcium-fortified orange juice to produce the final molarities listed in Table 1. The anodization reaction cell consisted of a 500 mL beaker of each electrolyte, the CPTi4 disc anode specimen, and two CPTi4 strip cathodes. Activated disc specimens were anodized using a DC rectifier (350 V, 10 A, Dynatronix, Amery, WI, USA) using a pulsed-galvanostatic waveform. Pulse current densities (700 mA/cm^2^, 29% duty cycle, frequency of 7.2 Hz) with a 40 ms on time/99 ms off time were applied for a period of 120 s in each electrolyte. We recently used the same anodization waveform in another hydroxyapatite-containing oxide study [40].

### 2.3. Oxide Surface Characterization

Thin-film X-ray diffraction (XRD, XDS 2000, Scintag, Franklin, MA, USA) was used to determine the crystalline phases present within the CPTi4 titanium substrate material and each of the anodized oxides. Representative specimens (*n* = 4) were rotated 1° away from the copper X-ray source (0.154 nm Cu-K_α_) to improve the interaction volume of X-rays. The XRD scans were performed over a 20° to 80° two-theta range. Optical imaging (Keyence, Osaka, Japan, VHX-1000) and scanning electron microscopy (SEM, Zeiss, Jena, Germany, Supra 40) were used for characterizing the topography of representative specimen surfaces. SEM imaging was performed using a 12 kV accelerating voltage at 5000× to capture the surface topography at micron-level, while a lower 9 kV accelerating voltage was used at 75,000× to avoid surface charging effects. A 3D optical profilometer (Keyence, Osaka, Japan, VK-X3000) was used to measure the surface roughness values from three randomized areas of each oxide specimen. The average roughness (S_a_) values and peak-to-valley roughness (S_z_) values for each oxide were calculated using Gwyddion software from the 3D profiles. Energy dispersive X-ray spectroscopy (EDS, EDAX, Mahwah, NJ, USA, APEX EDS Software Suite) was used to collect the oxide surface chemistries using an accelerating voltage of 12 kV at 500×. Triplicate areas on each specimen were used to collect the representative EDS spectra. The average surface concentrations of each element and the respective surface Ca/P ratios were calculated for each oxide. Finally, attenuated total reflectance Fourier transform infrared spectroscopy (ATR FTIR) was used on representative specimens from each group to determine molecular differences. A Spectrum 100 FTIR (Perkin-Elmer, Waltham, MA, USA) was used over the range of 650–4000 cm^−1^ at a 100 cm^−1^ spectral resolution.

### 2.4. Oxide Cross-Sectional Characterization

Representative oxide specimens were cross-sectioned and mounted in a conductive epoxy (Polyfast, Struers, Cleveland, OH, USA), wet ground with 220 grit SiC paper, polished with a 9 µm diamond suspension, and then to a final surface finish in a 0.02 μm colloidal silica suspension (Struers, Cleveland, OH, USA). Ten cross-sectional images were collected from each oxide, and five thickness measurements were carried out on each image. This produced a combination of 50 cross-sectional thickness measurements per oxide. Triplicate EDS line scans were collected on each oxide using a 12 kV accelerating voltage and a magnification range of 5000–10,000× in order to determine the compositional variations throughout the oxide cross-sections. A scan resolution level of 10 data points per micron for thinner oxide layers and 3 data points per micron for thicker oxide layers was used to estimate the chemistry of the outermost oxide surfaces. We recently used these same oxide cross-sectional characterization methods in another hydroxyapatite-containing oxide study [40].

### 2.5. Oxide Adhesion Quality

The VDI 3198 standard Rockwell C indentation test was used for the evaluation of the adhesion quality of all the anodized oxides [42]. This is an indentation-based adhesion strength assessment and has been used frequently in other implant coating studies [24,27,43,44]. Three indentations were performed on representative specimens from each of the oxides using a Rockwell C indenter with a load value of 150 kg, followed by imaging using an optical microscope. The obtained images were compared to the VDI 3198 standard adhesion quality maps [24,27,42,43,44].

### 2.6. Oxide Microhardness Assessment

The anodized oxide microhardness was evaluated using a Vicker’s microhardness tester (Clark CM-400AT, Sun-Tec, Novi, MI, USA) with a 300 g load and a dwell time of 12 s (HV 0.3). Ten indentations were placed into representative oxide specimens and evaluated. This microhardness method has also recently been used in another anodization study [24].

### 2.7. XPS Surface Analyses

X-ray photoelectron spectroscopy (XPS, Thermo Scientific, Waltman, MA, USA, K-Alpha XPS System Avantage v5.9911 Software Suite) characterization was also performed to further characterize the outermost surface layer of the hydroxyapatite-containing oxide. The XPS system, having a monochromatic X-ray source at 1486.6 eV, conforming to the Al K_α_ line, and an X-ray power of 75 W at 12 kV with a spot size of 400 μm^2^, was used for all experiments. The base pressure of the K-Alpha instrument was at 9.0 × 10^−10^ mbar. The instrument was calibrated to give a binding energy of 84.0 eV for Au 4f_7/2_ and 284.8 eV for the C1s line of aliphatic carbon present on the non-sputtered samples. The photoelectrons were collected at a take-off angle of 90° relative to the sample surface, and a series of XPS spectra were performed in the constant analyzer energy mode. Triplicate survey spectra were collected from different areas of a representative specimen (*n* = 1) using a pass energy of 200 eV and a 1.0 eV step size. Triplicate high-resolution (HR) core level spectra of C 1s, O 1s, P 2p, Mg 1s, Ca 2p, and Ti 2p were also taken using a 40 eV pass energy, 0.1eV step size, and an average of 50 scans.

### 2.8. Ion Release Rates

Inductively coupled plasma optical emission spectrometry (ICP-OES, SPECTRO AMETEK, SPECTROGREEN, Kleve, Germany, SPECTRO ICP Analyzer Pro Software) was used to determine the release profiles and cumulative release profiles of Ca and magnesium (Mg) ions from the hydroxyapatite-forming oxide group. Representative anodized disc specimens (*n* = 3) were immersed in 10 mL of calcium- and magnesium-free phosphate-buffered saline (PBS) solution (MP Biomedicals, Santa Ana, CA, USA). The specimens were transferred to fresh PBS at time points 2, 4, 6, 8, 10, 12, 14, 18, 22, and 30 days. Then, 300 μL of nitric acid and 50 μL of hydrochloric acid were added to each collected solution to dissolve any larger particulate matter. The solutions were then filtered using 0.4 μm syringe filters and analyzed for Ca and Mg release concentrations in parts per million (ppm).

### 2.9. Statistical Analyses

Welch’s one-way ANOVA (α = 0.05) with *post hoc* Dunnett’s T3 analyses were used to determine significant differences in surface roughness values and the cross-sectional thickness values amongst all the oxide groups due to the observance of unequal variance within the datasets.

## 3. Results

### 3.1. General Oxide Surface Characterization

#### 3.1.1. Oxide Crystallinity Analyses

**Figure 1:**
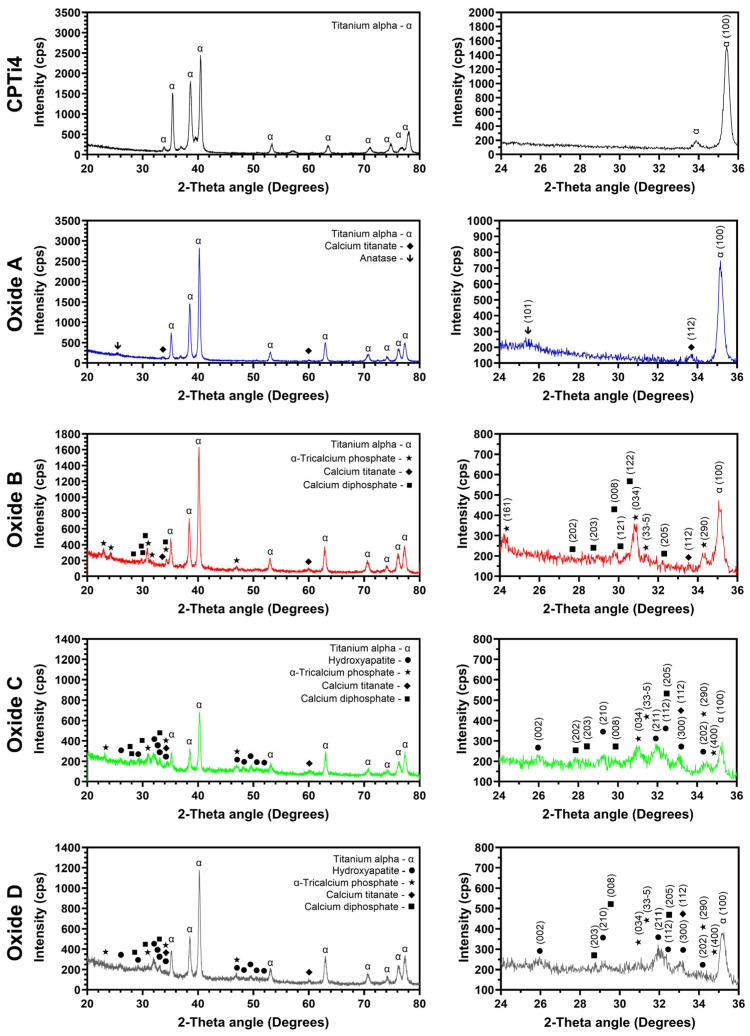
Representative X-ray diffraction scans of the CPTi4 substrate material and each oxide. Full ranges of 20° to 80° two-theta scans are shown on the left side. A zoomed-in 24° to 36° two-theta angle region is shown to the right side to emphasize the formation of crystalline calcium oxide phases. The anatase phase was formed in oxide A. Calcium titanate and calcium diphosphate phases were formed in all the oxides. Oxides C and D showed a combination of α-tricalcium phosphate and hydroxyapatite phases, with oxide D having predominantly hydroxyapatite.

The representative crystallinity results from the CPTi4 substrate material and each anodized oxide are provided in Figure 1. Furthermore, 20° to 80° two-theta scans are shown to the left side of Figure 1 to show all crystalline phases that are present. A zoomed-in 24° to 36° two-theta region is also provided to the right side of Figure 1 to emphasize the formation of crystalline calcium compound phases. The CPTi4 substrate material and each oxide group exhibited alpha phase titanium peaks. The oxide groups also exhibited a transition in crystallinity from oxides A through D evolving through various calcium compounds. Oxide A showed anatase phase titanium dioxide and calcium titanate (CaTiO_3_) formation, whereas oxide B revealed predominantly α-tricalcium phosphate with calcium diphosphate and calcium titanate phases. Oxides C and D showed a combination of calcium diphosphate, calcium titanate, α-tricalcium phosphate, and hydroxyapatite phase formation. The relative intensities of calcium diphosphate and α-tricalcium phosphate were shown to decrease in transitioning from oxides B, C, and D. Oxide C was the first to show hydroxyapatite formation in combination with calcium diphosphate and α-tricalcium phosphate, while oxide D showed predominantly hydroxyapatite.

#### 3.1.2. Oxide Surface Topographies

**Figure 2:**
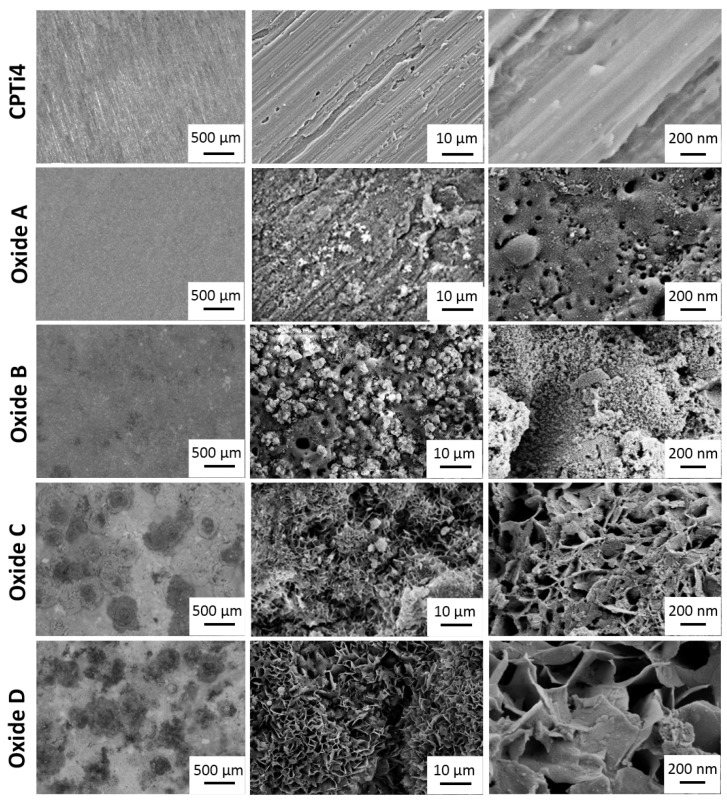
Optical microscopy and SEM images (left column—100× optical images, middle column—SEM 5000× micro-scale topography, right column—SEM 75,000× nano-scale topography) for CPTi4 substrate material and each anodized oxide. Oxide A exhibits a uniform greyish-white appearance with nano-scale porosity and small white deposits. Oxide B shows increased white deposits with micro- and nano-scale porosity. Oxides C and D exhibit micro- and nano-scale petal-like structures.

Oxide optical microscopy and SEM images are compiled in Figure 2. Low-magnification optical microscopy images are shown in the left column of Figure 2, and SEM images of the micro and nano-scaled surfaces are provided in the rightmost two columns of Figure 2. The CPTi4 un-anodized specimens showed evident markings remaining from the cutting and grinding specimen preparation. For oxide A, optical images revealed a uniform greyish-white appearance, and micron-scaled SEM images showed dispersed small white deposits across the surfaces. Nanoscale SEM images for oxide A exhibited surface nanopores. The optical images for oxide B revealed somewhat larger white deposits across the surfaces and some small localized darker areas. Micron-scaled oxide B images revealed pores in the oxide layer and a significant increase in the white deposits compared to those for oxide A. Nanoscale SEM images for oxide B showed fine white deposits uniformly distributed across the oxide surfaces. Optical images for oxide C revealed an increase in larger localized white deposits and some dark deposits remaining on the surfaces. Micron-scaled SEM images showed a significant morphology change to petal-like features while some white deposits remained visible. It should be noted that oxide surface pores were no longer visible at this scale. Nanoscale SEM images again showed petal-like surfaces with some nano-scale porosity still present. Optical images for oxide D revealed a similar distribution of white and dark deposits as compared to oxide C. Micron-scaled SEM images showed petal-like surfaces with very few remaining white deposits. Similar to oxide C, the surface pores were no longer visible in the micron-scale oxide surfaces. Nanoscale SEM images also showed larger petal-like surface features but without the remaining nano-scale porosity.

#### 3.1.3. Oxide Surface Roughness

**Figure 3:**
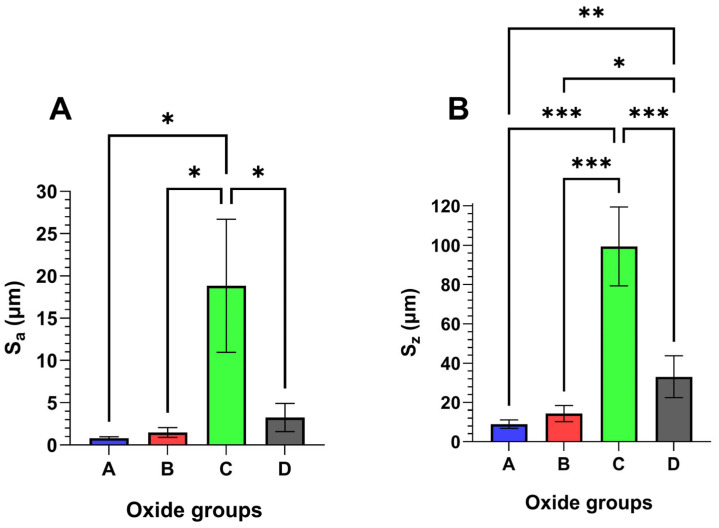
Representative 3D optical profilometer oxide surface roughness values. Oxide C was found to have significantly higher (**A**) oxide average surface roughness (S_a_) values (*p* < 0.05) and (**B**) oxide peak-to-valley roughness (S_z_) values (*p* < 0.001) compared to the other oxides. Oxide D also had significantly higher S_z_ values compared to oxide A (*p* < 0.01) and oxide B (*p* < 0.05). The * symbol denotes a statistical significance of *p* < 0.05. the ** symbols denote a statistical significance of *p* < 0.01, and the *** symbols denote a statistical significance of *p* < 0.001.

Representative oxide surface roughness results are compiled in Figure 3. Average oxide surface roughness, S_a_, values are shown in Figure 3A, while peak-to-valley roughness, S_z_ values, are compiled in Figure 3B. Oxide C was shown to exhibit significantly higher average S_a_ (*p* < 0.05) and S_z_ (*p* < 0.001) values compared to the other oxides. Additionally, Oxide D had significantly higher S_z_ values compared to oxide A (*p* < 0.01) and oxide B (*p* < 0.05).

#### 3.1.4. Oxide Surface Compositions

**Figure 4:**
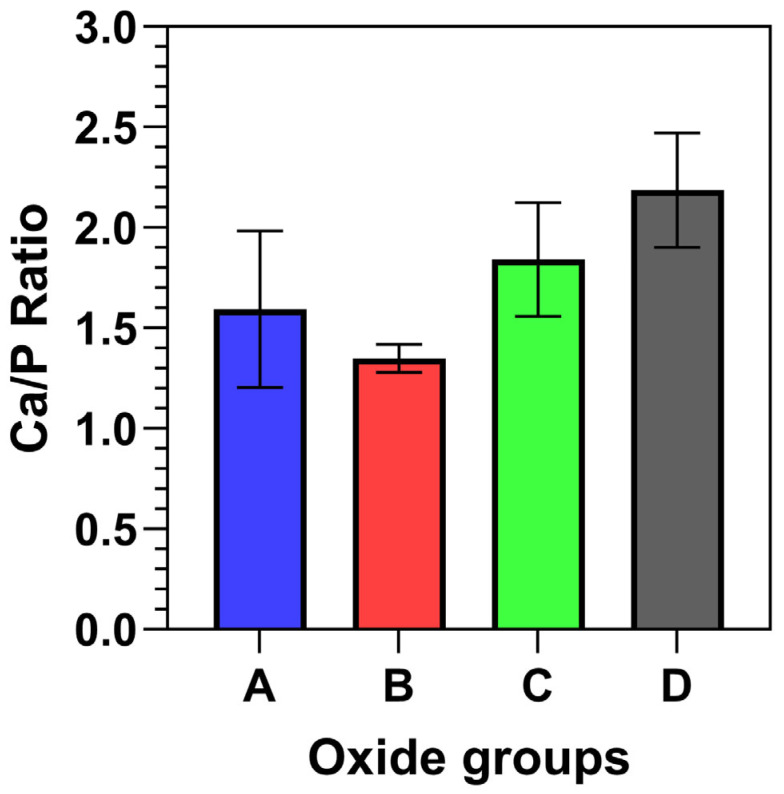
Representative oxide surface Ca/P ratios. The EDS-derived surface Ca/P ratios were shown to increase with the changing anodization electrolytes.

The EDS surface compositions for each oxide group are compiled in Table 2. The corresponding oxide average Ca/P ratios are provided in Figure 4. Each oxide group showed the presence of titanium (Ti) and oxygen (O), with the amount of Ti decreasing in transitioning from oxide A through D. For Oxide A, 4% Ca and 3% P and some Mg (<1%) dopants were incorporated. Oxide B showed substantially higher dopant uptake, with approximately 13% Ca, 9% P, and <1% Mg. Oxides C and D exhibited similar dopant uptake levels of at least 10% Ca, 5% P, and <1% Mg. The surface EDS-derived Ca/P ratios were also shown to generally increase with the transitions from oxide A to oxide D.

#### 3.1.5. Oxide Molecular Structure Analyses

**Figure 5:**
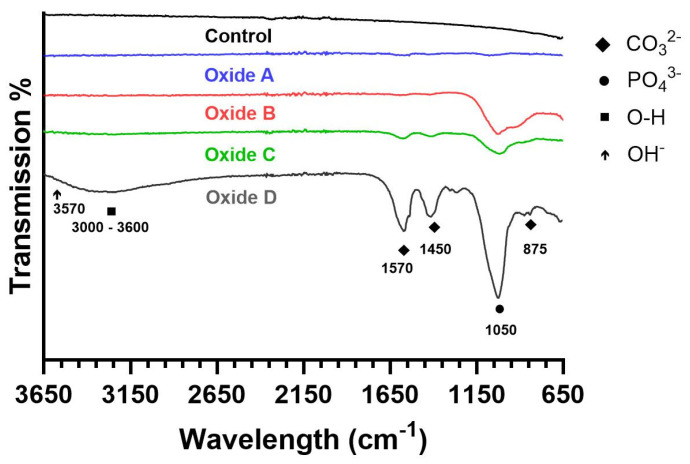
Representative FTIR spectra. The CPTi4 substrate and Oxide A lack definitive peaks, while oxides B, C, and D exhibit a strong PO_4_^3−^ peak at 1050 cm^−1^. The oxide D exhibits a more pronounced peak. Broad O-H bands (3000–3600 cm^−1^) are observed in oxide D and attributed to adsorbed water molecules. Oxides C and D show CO_3_^2−^ substitution peaks at wavelengths of 875, 1450, and 1570 cm^−1^ confirming the formation of bone-like carbonated apatite.

Representative FTIR scans for the CPTi4 substrate material and each oxide group are compiled in Figure 5. The CPTi4 substrate material showed no visible FTIR peaks. Oxide A had some poorly defined absorption peaks around 1050 cm^−1^, 1450, and 1570 cm^−1^. Oxides B, C, and D showed intensive absorption peaks at 1050 cm^−1^, corresponding to phosphate (PO_4_^3−^) groups, indicating the presence of calcium phosphate compounds formed on the surfaces of the respective oxides, as shown in Figure 1 [24,45]. Additionally, oxide B also had some poorly defined carbonate (CO_3_^2−^) (1450 and 1570 cm^−1^) peaks, whereas oxides C and D showed well-defined characteristic absorption peaks for CO_3_^2−^ (875, 1450, and 1570 cm^−1^) [24,45,46]. These peaks are more pronounced in oxide D compared to oxides B and C. Furthermore, a broad O-H band between 3000–3600 cm^−1^ is evident in oxide D and is attributed to the bending mode of adsorbed water molecules [24,45,46]. However, the characteristic hydroxyapatite OH^−^ peak at 3570 cm^−1^, found in synthetic hydroxyapatite coatings, is absent in the oxides C and D [45,46]. The presence of CO_3_^2−^ substitution peaks (875, 1450, and 1570 cm^−1^) instead of the characteristic hydroxyapatite peak (3570 cm^−1^) confirmed the formation of bone-like carbonated apatite [24,45,46]. Oxide D exhibits more pronounced high-intensity peaks at these positions, indicating higher degrees of carbonate substitution compared to the oxide C counterparts.

### 3.2. Oxide Cross-Section Evaluation

#### 3.2.1. Oxide Thickness Evaluation

**Figure 6:**
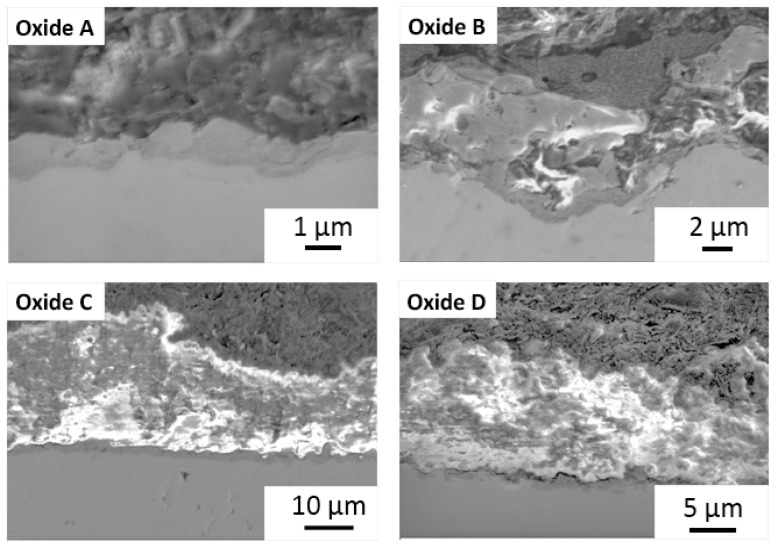
Representative SEM cross-sectional images of each oxide.

**Figure 7:**
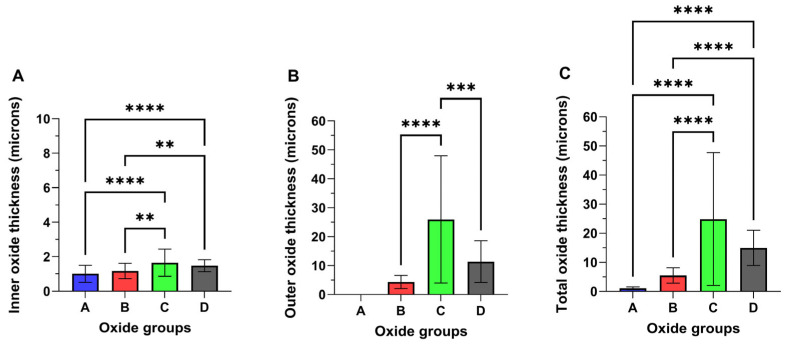
Representative oxide cross-sectional thickness values: (**A**) Inner layer oxide and (**B**) outer layer oxide. (**C**) Total oxide. Oxides C and D show significantly thicker inner oxide layers (1.5 µm) compared to Oxide A (*p* < 0.0001) and Oxide B (*p* < 0.01). Among the bi-layered oxides, oxide C has the thickest outer layer (26 µm), significantly greater than Oxide D (14 µm) (*p* < 0.001) and oxide B (4 µm) (*p* < 0.0001). Oxides C (27.6 µm) and D (15 µm) have the highest total oxide thickness, significantly greater than Oxide B (5.5 µm) (*p* < 0.0001) and Oxide A (1 µm) (*p* < 0.0001). The ** symbols denote a statistical significance of *p* < 0.01. the *** symbols denote a statistical significance of *p* < 0.001, and the **** symbols denote a statistical significance of *p* < 0.0001.

Representative cross-sectional images of each oxide are provided in Figure 6, and the measured oxide cross-sectional thickness values are compiled in Figure 7. Oxide A showed a single layer oxide and Oxides B, C, and D were found to exhibit bi-layered oxides as shown in Figure 6 and Figure 7. The analyses of the inner and outer layer portions of the oxide thicknesses were evaluated separately, as compiled in Figure 7A,B. The total oxide thickness for each oxide group is compiled in Figure 7C. Interestingly, Oxides C and D revealed significantly thicker inner oxides of approximately 1.5 μm compared to 1.2 μm for oxide B (*p* < 0.01) and 1 μm for oxide A (*p* < 0.0001). Of the bi-layered oxides, oxide C revealed a significantly thicker outer layer of approximately 26 µm, compared to 14 µm for oxide D (*p* < 0.001) and approximately 4 µm for oxide B (*p* < 0.0001). Similarly, oxides C and D revealed significantly thicker total oxide thickness values of 27.6 µm and 15 µm compared to 5.5 µm for oxide B (*p* < 0.0001) and 1 um for oxide A (*p* < 0.0001).

#### 3.2.2. Oxide Cross-Sectional Compositional Analyses

**Figure 8:**
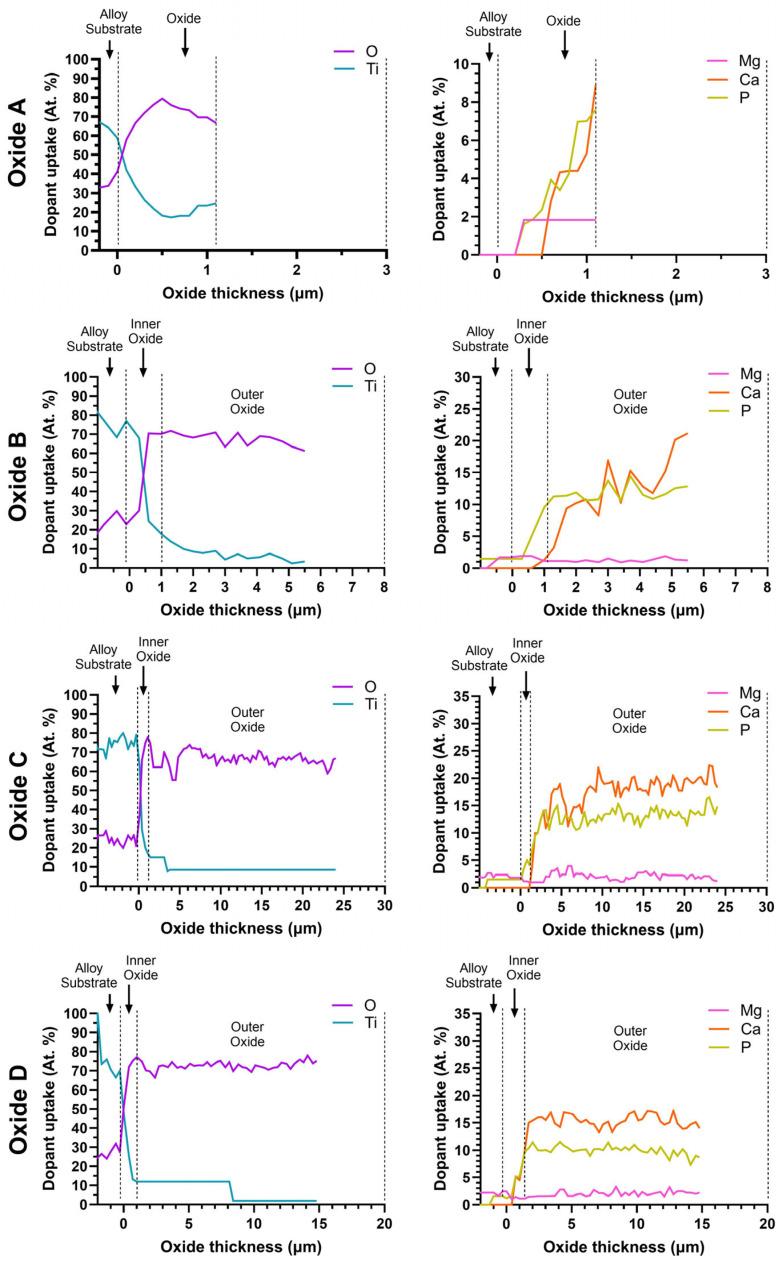
Representative EDS line scans of oxide dopant distributions across the layer cross-sections. The Ti and O element distributions are provided in the left column, and distributions of the Ca-, P-, and Mg-dopant elements incorporated from the anodization electrolyte are provided in the right column. The substrate interface is located on the left side of each window, followed by the inner and outer oxides moving left to right. Oxide A revealed a single layer oxide structure consisting of Ti, O, Ca, P, and Mg. Oxides B, C, and D revealed a bi-layered oxide structure, with the concentration of Ti decreasing from the substrate toward the outer oxide. Ca-, P-, and Mg-dopant uptake begins in the inner oxide layer and continues to increase before reaching a relatively stable level in the outer oxide for oxides B, C, and D. X-axis scales are adjusted to best represent the oxide thickness.

Figure 8 shows representative line scans for each oxide cross-section in order to evaluate the distribution of the incorporated elements. For clarity in the complex figure, the distribution of elements across the oxide cross-sections was divided into two sub-groups. Ti and O distributions are shown in the left column, while the Ca-, P-, and Mg-dopant elements are shown in the right column. Each window of Figure 8 shows the substrate material/oxide interface to the left side, followed by the inner and outer layer oxides moving left to right. The cross-sectional EDS line scans confirmed the bi-layered structures for the oxides B, C, and D. The inner oxide layers for each oxide were titanium-dioxide-rich. Since Oxide A was only a single layer, Ca, P, and Mg dopants were also present within this same oxide layer. In contrast, the Ca, P, and Mg dopants were predominately incorporated into the outer oxide layers in oxides B, C, and D. The concentrations of Ti in the outer layers of the B, C, and D oxides were substantially reduced compared to that in the same inner layer oxides. Thus, the outer layers in Oxides B, C, and D correspond to the calcium phosphate compounds, including tricalcium phosphate and hydroxyapatite, which were confirmed in the XRD analyses in Figure 1.

**Figure 9:**
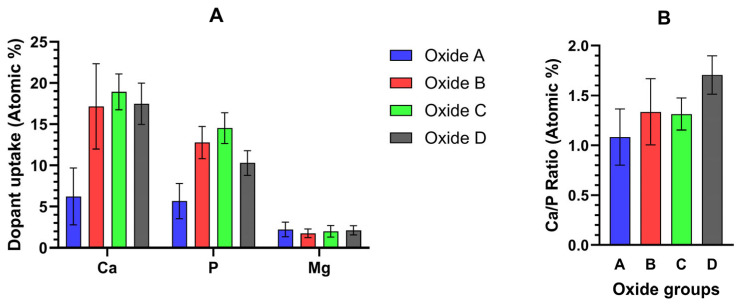
(**A**) EDS-derived Ca-, P-, and Mg-dopant uptake levels from the outermost 0.5 microns for oxide A and the outermost 2 microns of oxides B, C, and D. (**B**) Corresponding EDS Ca/P ratios within the outermost oxide surfaces. Ca/P surface ratios range from 1.1 to 1.7 for the oxides.

EDS Ca-, P-, and Mg-dopant uptake levels within the outermost 0.5 microns of oxide A and the outermost 2 microns of Oxides B, C, and D are compiled in Figure 9A. The corresponding oxide Ca/P ratios are compiled in Figure 9B. The corresponding Ca/P ratio from these outermost layers of each bi-layered oxide ranged from 1.3 to 1.7, which is much closer to the known ranges for tricalcium phosphate and hydroxyapatite compounds.

### 3.3. Oxide Layer Adhesion Results

**Figure 10:**
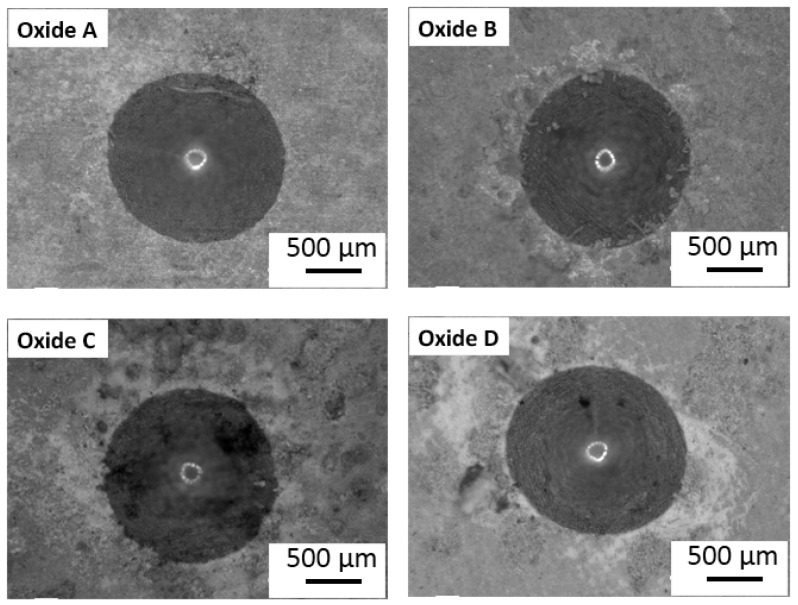
Oxide layer adhesion quality results for each oxide. Oxide B revealed some microcracking and delamination. The oxides A, C, and D did not exhibit any delamination, while Oxides C and D exhibited minor microcracking.

Representative VDI 3198 standard oxide layer adhesion results are compiled in Figure 10. The oxide adhesion results for oxide B revealed some microcracking and delamination. However, the oxide layer formed in oxides A, C, and D showed good adhesion to the titanium substrate with no evidence of delamination in either oxide, but mild microcracking was observed in oxides C and D.

### 3.4. Summary of Oxide Characteristics

Given the large number of research techniques used in this study, we have created a summary table of the oxide characteristics in Table 3.

### 3.5. Additional Characterization of Oxide D

Since oxide D predominately contained the hydroxyapatite phase within the outermost oxide layer, additional microhardness and XPS characterizations of this oxide group were performed. Ca and Mg release profiles from these promising oxide D surfaces were also produced.

#### 3.5.1. Oxide Microhardness

**Figure 11:**
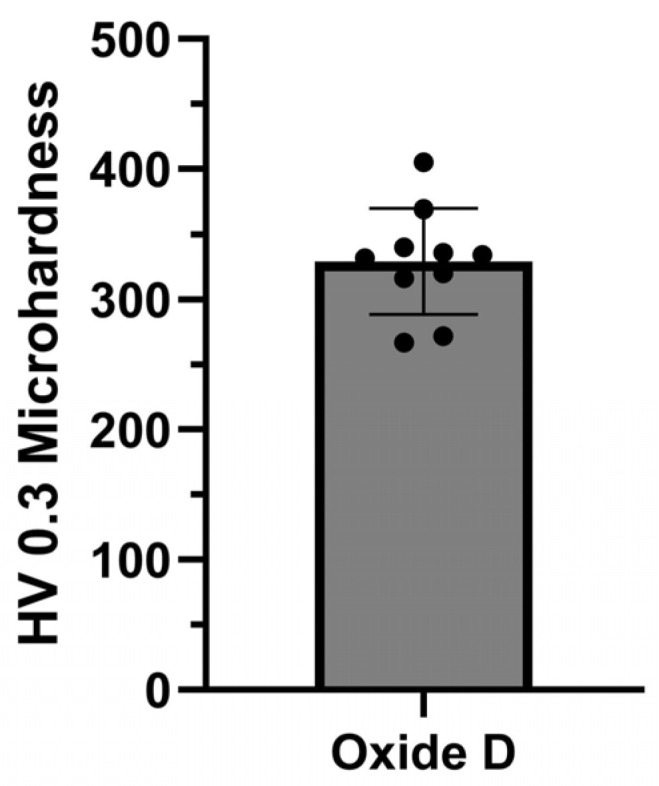
Oxide D microhardness evaluation.

The Vicker’s microhardness dataset generated for oxide D is provided in Figure 11. The individual indentation values are included within the bar chart containing the average and standard deviation HV 0.3 values. The ten microhardness indentations exhibited HV 0.3 values of 329.2 ± 40.9.

#### 3.5.2. XPS Surface Chemistry

**Figure 12:**
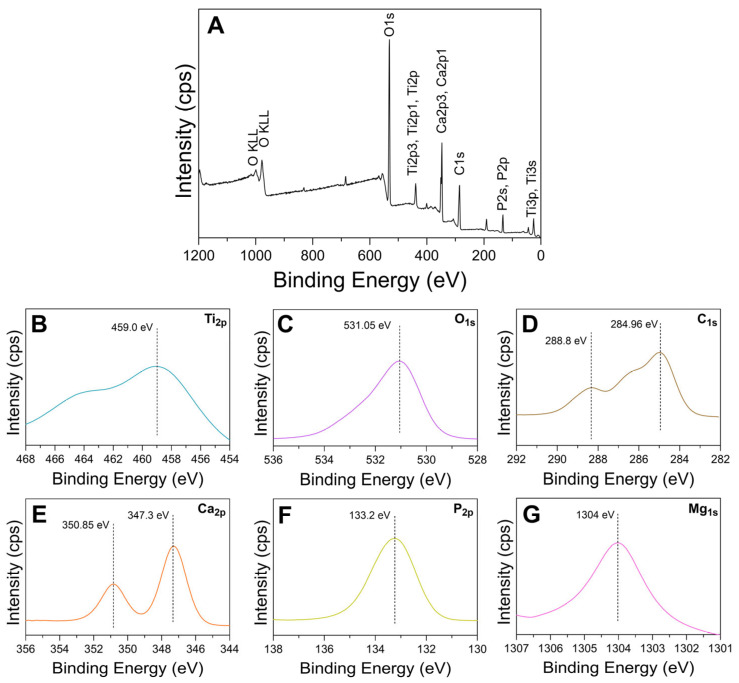
Representative XPS spectra for oxide D. (**A**) survey full spectra, (**B**) Ti_2p_, (**C**) O_1s_, (**D**) C_1s_ (**E**) Ca_2p_, (**F**) P_2p_, and (**G**) Mg_1s_.

Representative XPS spectra for oxide D are shown in Figure 12. XPS survey spectra revealed Ti, O, Ca, P, Mg, and C peaks, as shown in Figure 12A. Dashed lines are included in the high resolution images in Figure 12 to denote specific peak positions to enable comparisons to other studies. The high-resolution spectra for the Ti_2p_ peaks are shown in Figure 12B. The Ti_2p3/2_ peaks with a binding energy of 459 eV represent the formation of TiO_2_ [47,48]. The O_1s_ peaks with a binding energy of 531.05, as shown in Figure 12C, are representative of calcium-phosphate-containing oxides [48,49]. The C_1s_ peaks exhibited approximately 284.96 eV and 288.8 eV binding energies, as shown in Figure 12D, and indicate the presence of C-C bonds and carbonates [47,49]. This finding is in good agreement with the carbonate substitution peaks found in oxide D FTIR analysis (Figure 5). The 284.96 eV binding energy peaks for C_1s_ are suggestive of carbon from surface contamination and may also be due to the presence of organics from the calcium-fortified orange juice component of the electrolyte [19,50]. The Ca_2p3/2_ and Ca_2p1/2_peaks revealed binding energies of approximately 347.3 eV and 350.85 eV, as shown in Figure 12E, and are indicative of the formation of calcium titanate (CaTiO_3_) and calcium phosphates [47,48,49,51,52,53,54]. The P_2p_ peaks exhibited binding energies of 133.6 eV, as shown in Figure 12F, indicative of phosphorus-containing titanium oxides and phosphates [47,49,51]. Finally, the Mg_1s_ peaks exhibited binding energies of 1303.9 eV, as shown in Figure 12G, which represents the presence of Mg-containing metal oxides [55].

#### 3.5.3. Calcium and Magnesium Ion Release Profiles

**Figure 13:**
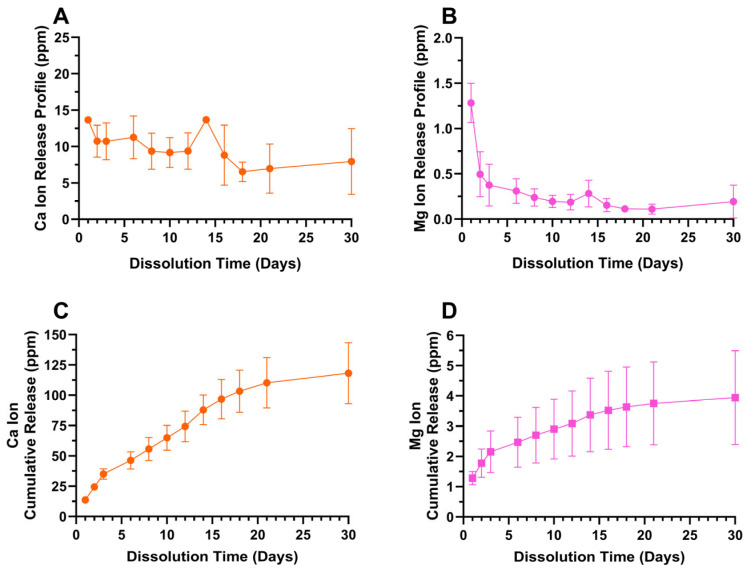
Oxide D (**A**) Ca ion release profile, (**B**) Mg ion release profile, (**C**) Ca ion cumulative release profile, (**D**) Mg ion cumulative release profile. Ca and Mg ion release profiles showed an initial burst release followed by a gradual release, indicating a sustained cumulative release over a period of 30 days. The Ca ion release reached a cumulative amount of approximately 120 ppm, whereas the Mg release was much lower at approximately 4 ppm over a period of 30 days.

ICP-OES derived 30-day Ca and Mg ion release profiles from the predominately hydroxyapatite-containing oxide D are provided in Figure 13. Both Ca and Mg ions showed a sustained release over the entire 30-day duration with an initial burst release. The Ca ion release reached a cumulative amount of approximately 120 ppm, whereas the Mg release was much lower at approximately 4 ppm over a period of 30 days.

## 4. Discussion

Hydroxyapatite and tricalcium phosphate are important calcium phosphates found in bone and have been widely used to form surface coatings on titanium implants to improve osseointegration [32,35]. Previously, α-tricalcium phosphate coatings on titanium implants created by magnesium-sputtering have increased BIC and peri-implant bone volumes in rabbit femurs [56]. Hydroxyapatite-coated titanium implants have a long history of use in dental and orthopedic implants. These coatings have shown improved osseointegration abilities both in vitro and in vivo. Hydroxyapatite implant coatings have previously been shown to have superior osteoblast cell viability as well as enhanced differentiation and mineralization [22,24,27,57]. These coatings have demonstrated early new bone induction surrounding the implant and improved BIC in rat and rabbit femur models in comparison to non-anodized implants [57,58]. However, the conventional techniques used for forming these coatings, like plasma spraying, have certain drawbacks, such as requiring specialized and expensive processing equipment, long preparation times, and lower than desirable adhesion strengths [37,38,39]. In the present study, we used an electrolyte combining calcium-fortified orange juice with monobasic calcium phosphate and calcium acetate. Interestingly, calcium acetate and monobasic calcium phosphate are also both commonly used as food stabilizers and leavening agents in baking products [59,60].

The anodization oxide groups formed in the present study revealed distinct transitions in terms of crystallinity, topography, molecular structure, chemistry, and cross-sectional composition with changes in the citrus-fruit-based electrolyte chemistry (Table 3). Oxide A showed crystalline compounds anatase and calcium titanate compounds within the single-layered oxide. Early CaP oxide studies showed oxide surface Ca/P ratios within the range of human bone and hydroxyapatite [27,61]. However, the same early CaP oxide studies showed difficulty forming crystalline hydroxyapatite during subsequent in vitro bioactivity testing [27,61,62]. It was later discovered that the formation of calcium titanate compounds during anodization facilitated the formation of hydroxyapatite during subsequent bioactivity testing [27,62,63,64,65,66,67]. Adding additional calcium acetate into the electrolyte resulted in the formation of α-tricalcium phosphate compounds in oxide B. Further increases of the calcium acetate concentrations in the electrolyte resulted in oxides C and D forming combinations of α-tricalcium phosphate and hydroxyapatite. While some research groups have formed hydroxyapatite-containing anodization coatings on titanium, only a few have formed the combination of hydroxyapatite, tricalcium phosphate, and calcium titanate, as observed in the present study [20,22,24,27]. Additionally, it has been suggested that tricalcium phosphate is more bioresorbable and degrades first, leaving surface porosity, which is subsequently used by calcium titanate to promote further bone growth [68]. In addition to anodization studies, a sol-gel study revealed that calcium titanate in the interlayer between the implant substrate and the hydroxyapatite-coated layer improved the adhesion strength between the two layers [69].

The surface topography of the implants plays an important role in its interaction with bone cells. The surface topographies of each citrus-fruit-based oxide group exhibited multiscale micro- and nano-scale features (Figure 2). Since each oxide was anodized using the same galvanostatic pulsed anodization process, the electrolyte chemistry changes were responsible for the changes in surface topography between the different oxide groups. Increases in the electrolyte calcium acetate concentration in oxides C and D correlated with an oxide surface topography transition to micro- and nano-scaled petal-like surface features. While the petal-like hydroxyapatite topography formation has been reported in some other anodization studies that formed hydroxyapatite, the combinations of white particle deposits and the petal-like hydroxyapatite structures shown in the fruit-based oxides C and D within the present study have not been previously reported elsewhere to the knowledge of the authors [20,22,46].

FTIR analyses on oxide A in the present study revealed some poorly defined absorption peaks. In contrast, oxide B revealed small absorption peaks for PO_4_^3−^ (1050 cm^−1^) in agreement with the α-tricalcium phosphate formation shown in Figure 1 in addition to some poorly defined CO_3_^2−^ (1450 and 1570 cm^−1^) peaks. Oxides C and D showed characteristic absorption peaks for PO_4_^3−^ (1050 cm^−1^) and CO_3_^2−^ (875, 1450, and 1570 cm^−1^), which agreed with the combinations of α-tricalcium phosphate and hydroxyapatite shown in the XRD analyses. Therefore, the oxides C and D confirmed the presence of carbonated or bone-like apatite, which was more prominent in oxide D.

Previous papers have mentioned that an electrolyte pH range of 5–6, while some others reported a pH range of 9–11, is needed for the formation of hydroxyapatite on the surface [63,70,71]. In contrast, the pH of the fruit-based electrolytes that formed oxides C and D in the present study was substantially more acidic, averaging 4.5 for oxide C and 4.3 for oxide D. These more acidic electrolyte pH ranges may have also contributed to the unique oxide topographies and structures shown in these novel oxides.

Some research groups have successfully been able to form bi-layered hydroxyapatite coatings on titanium surfaces directly using a single-step anodization process [20,22,24,27]. Some of these oxides demonstrated improved adhesion strengths between the titanium substrate and the hydroxyapatite coatings than those of conventional hydroxyapatite spray coatings due to the interlocking at the interface between the inner titanium oxide layer and the outer hydroxyapatite layers [20,24]. Similarly, the oxides B, C, and D in the present study were shown to form a bi-layered oxide consisting of a titanium-dioxide-rich inner layer and a tricalcium phosphate/hydroxyapatite-rich outer layer. This was further reflected in the oxide D XPS analysis, wherein the TiO_2_ peak exhibited lower intensities, indicating the presence of an outer layer. Oxide B revealed some microcracking and delamination, indicating inadequate coating adhesion strengths, potentially due to the amorphous nature of the predominant α-tricalcium phosphate phase present in the oxide [72]. However, oxides A, C, and D showed good adhesion to the titanium substrate, as evidenced in the VDI indentation testing. Oxide D also showed HV 0.3 microhardness values that were comparable to a previous study on hydroxyapatite-containing anodized oxides [24].

Like previous CaP anodization oxide studies that formed hydroxyapatite, our study also showed significant uptake of Ca and P into each oxide layer, as evidenced by the EDS surface analyses [20,21,22,23,24,25,26]. The average EDS-derived surface Ca/P ratios for oxides A and C were found to be in the range of bone and hydroxyapatite (1.5–1.7) [61,73]. Oxide B revealed a surface Ca/P ratio slightly below 1.5, while oxide D revealed an average surface Ca/P ratio slightly over 2.0. However, surface EDS analysis results are known to average values from a substantial interaction volume of the SEM electron beam with the subject material. The below-surface depth and lateral dimension of this interaction volume depend greatly on the accelerating voltage used, as well as the elements present and crystalline structures present in the subject material. In contrast, the use of cross-sectional EDS line scan analyses of these oxides facilitates a clearer examination of the actual Ca/P ratios shown near the surface of these coatings because the below-surface depth interaction volume is more consistent. An examination of the data from the outermost two microns of the B, C, and D oxides revealed oxides B and C to exhibit a Ca/P ratio below 1.5, while oxide D exhibited a ratio of 1.7. Thus, the oxide D ratio showed very good agreement with the known 1.67 stoichiometric ratio of hydroxyapatite [61,73]. Another recent study revealed that α-tricalcium phosphate, having Ca/P ratios below 1.5, appeared as 1–2 μm dense agglomerates under SEM, similar to the deposits observed in oxides B and C in the present study [74]. Therefore, the EDS analysis of these near oxide surface areas from oxides C and D in the present study helps to elucidate the greater hydroxyapatite formation shown within oxide D. Since the outermost two microns of the oxide would likely be in constant contact with bodily fluids and tissues, this was an exciting result.

Each citrus-fruit-based oxide in the present study also showed some amount of Mg uptake. This was initially a somewhat surprising result since Mg was not intentionally added other than the amounts contained within the calcium-fortified orange juice component of the anodization electrolyte. Mg is one of the topmost abundant elements in the body and the most prevalent in cells [75]. In its ionic form, Mg plays a vital role in bone growth and cellular functions such as proliferation, signaling, and metabolism [75]. Mg has also been shown to inhibit osteoclast differentiation and bone resorption, promoting bone growth and regeneration [76,77]. Mg-doped titanium surfaces revealed enhanced promotion of osteogenesis, cell adhesion, and angiogenesis through pathways like PI3K, ERK, and BMP-4 [78,79]. A few previous studies have successfully incorporated Mg dopants into anodized oxides [78,80,81]. Mg-incorporated anodized titanium surfaces enhance osteogenic differentiation and rapid bone integration, as evidenced by increased removal torque values in animal models [78,81]. Moreover, coatings combining Mg and hydroxyapatite have shown improved adhesion, trabecular bone formation, and osseointegration, with superior interfacial strength and push-out forces compared to hydroxyapatite-only coatings [82]. Oxide D in the present study exhibited a Mg-doped predominantly bone-like carbonated hydroxyapatite outer oxide structure. Furthermore, a sustained Mg release was shown over a 30-day period in our ICP-OES study for oxide D. This oxide coating shows much promise to improve the osseointegration characteristics of future implants.

The presence of Ca and Mg ions surrounding the implant microenvironment play a critical role in enhancing osseointegration [78,83]. Ca ions promote fibrin clot formation, which serves as a chemotactic scaffold for recruiting osteogenic cells to the implant site, further supporting cell attachment, proliferation, and differentiation, thereby accelerating the bone healing and remodeling process [28,29]. Additionally, Ca ions contribute to the later stages of the coagulation cascade, reducing complement activation and fostering a more favorable environment for bone regeneration during the healing phase [28,29,30]. Mg ions, on the other hand, activate the transient receptor potential melastatin 7 (TRPM7)/phosphoinositide 3-kinase (PI3K) signaling pathway, which induces osteogenic differentiation in osteoblasts and mesenchymal stem cells [83]. Additionally, Mg ions promote the stabilization of β-catenin, which is essential for the early stages of mesenchymal stem cell osteogenic differentiation [83]. Recently, another research group studied the effects of Ca and Mg ion releases from titanium implant surfaces that were individually doped by these elements via wet chemical treatment [83]. Interestingly, it was found that the Mg-doped surfaces showed earlier cell spreading, focal adhesion, and osteogenic differentiation of the primary bone marrow mesenchymal stem cells compared to the Ca-doped surfaces. In terms of ion release, this study revealed Ca-ion concentrations to be about 3.6 ppm on day 1, with cumulative concentrations reaching 7.3 ppm by day 8, whereas that for Mg was 0.7 ppm by day 1 and 1.9 ppm by day 8. In the present study, the Ca-ion release was significantly higher, with concentrations of about 14 ppm on day 1 and a cumulative release of about 56 ppm by day 8. Whereas for Mg ions, it was 1.3 ppm on day 1 and 2.7 ppm by day 8. Additionally, the present study shows potential for a synergistic effect of prolonged Ca and Mg ion release over a period of 30 days. Thus, oxide D formed in the present study revealed a unique combination of calcium- and magnesium-releasing carbonated hydroxyapatite- and tricalcium-phosphate-containing surfaces, which show a strong potential for improving osseointegration.

One limitation of these promising study results would be the complexity of the commercially available calcium-fortified orange juice used as a component of the electrolyte. The proprietary commercially available orange juice composition may be difficult to replicate using only the information on the printed label. Additionally, even though the available literature on hydroxyapatite-containing oxides suggest the topographical, chemical, mechanical, and dissolution properties shown for oxide D look very promising for future cell culture and animal studies, the lack of in vitro and in vivo assessments of this coating somewhat limits the immediate applicability to current medical and dental implant technologies. Future studies are planned to address these limitations.

## 5. Conclusions

The citrus-fruit anodization process in the present study produced one predominately bone-like carbonated apatite oxide exhibiting good adhesion strengths in VDI indentation testing and HV 0.3 hardness values above 300. This promising oxide also exhibited sustained release of Ca and Mg over a 30-day period in the dissolution assessment. To the knowledge of the authors, this study was the first to form a combination of calcium- and magnesium-releasing carbonated hydroxyapatite and tricalcium phosphate oxides on titanium implant materials using a single-step anodization process in a citrus-fruit-based electrolyte. Given the importance of the hydroxyapatite coatings toward improving osseointegration of dental and orthopedic implants combined with the poor adhesion strengths of some hydroxyapatite coatings, resulting in delamination and potential loosening of implant devices, the novel oxides prepared our study show much potential to improve future patient outcomes with titanium implants.

## 6. Patents

This research work is a part of a provisional patent.

## Figures and Tables

**Table 1:** Composition of anodization electrolytes for forming calcium-rich oxides.

Group	Calcium-Fortified Orange Juice	Calcium Phosphate (M)	Calcium Acetate (M)
Oxide A	500 mL	-	0.15
Oxide B	500 mL	0.15	0.2
Oxide C	500 mL	0.15	0.25
Oxide D	500 mL	0.15	0.275

**Table 2:** EDS surface chemistry for each oxide group.

Elements	Group			
	Oxide A (At.%)	Oxide B (At.%)	Oxide C (At.%)	Oxide D (At.%)
Titanium	12 ± 4	4 ± 1	<1	<1
Oxygen	58 ± 4	60 ± 5	51 ± 3	47 ± 2
Calcium	4 ± 2	13 ± 3	10 ± 1	10 ± 2
Phosphorus	3 ± 1	9 ± 2	5 ± 1	5 ± 2
Magnesium	<1	<1	<1	<1

**Table 3:** Summary of characteristics of anodization oxide groups formed using citrus-fruit-based electrolytes.

Characteristics (Technique)	Groups			
	Oxide A	Oxide B	Oxide C	Oxide D
Crystallinity (XRD)	Anatase, calcium titanate	α-Tricalcium phosphate, calcium diphosphate, and calcium titanate	α-Tricalcium Phosphate, calcium diphosphate, calcium titanate, and hydroxyapatite	Predominantly hydroxyapatite with some α-tricalcium phosphate, calcium diphosphate and calcium titanate
Surface Topography (SEM)	Small white deposits with nanopores	Larger white deposits with nanopores	Small petal-like features with white deposits and some nanopores	Larger petal-like features with few white deposits and no nanopores visible
Molecular structure (FTIR)	Poorly defined PO_4_^3−^ peaks, some CO_3_^2−^ peaks	Well defined PO_4_^3−^ and poorly defined CO_3_^2−^ peaks	Well defined PO_4_^3−^ peaks, CO_3_^2−^ substitution peaks	Higher-intensity PO_4_^3−^ and CO_3_^2−^ substitution peaks
Oxide Surface Chemistry (SEM and EDS)	Ca: 4.4%, P: 2.7%, Mg: 0.1%	Ca: 12.8%, P: 9.4%, Mg: 0.2%	Ca: 9.8%, P: 5.4%, Mg: 0.1%	Ca: 9.9%, P: 4.7%, Mg: 0.1%
Oxide Cross-sectional Analyses (SEM and EDS)	Single layer; Predominantly Ti, O, Ca, P, and Mg	Bi-layered; outer layer predominantly Ca, P, and Mg	Bi-layered; outer layer predominantly Ca, P, and Mg	Bi-layered; outer layer predominantly Ca, P, and Mg
Oxide Adhesion (VDI)	Acceptable, No delamination and microcracking	Unacceptable, Delamination and Cracking	Acceptable, No delamination but minor microcracking	Acceptable, No delamination but minor microcracking

## Data Availability

The data that support the findings of this study are available from the corresponding author upon reasonable request.
